# *In vitro* reconstitution and characterization of pyruvate dehydrogenase and 2‐oxoglutarate dehydrogenase hybrid complex from *Corynebacterium glutamicum*

**DOI:** 10.1002/mbo3.1113

**Published:** 2020-08-30

**Authors:** Hirokazu Kinugawa, Naoko Kondo, Ayano Komine‐Abe, Takeo Tomita, Makoto Nishiyama, Saori Kosono

**Affiliations:** ^1^ Biotechnology Research Center The University of Tokyo Bunkyo‐ku Japan; ^2^ Collaborative Research Institute for Innovative Microbiology The University of Tokyo Bunkyo‐ku Japan; ^3^ RIKEN Center for Sustainable Resource Science Wako Japan

## Abstract

Pyruvate dehydrogenase (PDH) and 2‐oxoglutarate dehydrogenase (ODH) are critical enzymes in central carbon metabolism. In *Corynebacterium glutamicum*, an unusual hybrid complex consisting of *Cg*E1p (thiamine diphosphate‐dependent pyruvate dehydrogenase, AceE), *Cg*E2 (dihydrolipoamide acetyltransferase, AceF), *Cg*E3 (dihydrolipoamide dehydrogenase, Lpd), and *Cg*E1o (thiamine diphosphate‐dependent 2‐oxoglutarate dehydrogenase, OdhA) has been suggested. Here, we elucidated that the PDH‐ODH hybrid complex in *C*.* glutamicum* probably consists of six copies of *Cg*E2 in its core, which is rather compact compared with PDH and ODH in other microorganisms that have twenty‐four copies of E2. We found that *Cg*E2 formed a stable complex with *Cg*E3 (*Cg*E2‐E3 subcomplex) *in vitro*, hypothetically comprised of two *Cg*E2 trimers and four *Cg*E3 dimers. We also found that *Cg*E1o exists mainly as a hexamer in solution and is ready to form an active ODH complex when mixed with the *Cg*E2‐E3 subcomplex. Our *in vitro* reconstituted system showed *Cg*E1p‐ and *Cg*E1o‐dependent inhibition of ODH and PDH, respectively, actively supporting the formation of the hybrid complex, in which both *Cg*E1p and *Cg*E1o associate with a single *Cg*E2‐E3. In gel filtration chromatography, all the subunits of *Cg*ODH were eluted in the same fraction, whereas *Cg*E1p was eluted separately from *Cg*E2‐E3, suggesting a weak association of *Cg*E1p with *Cg*E2 compared with that of *Cg*E1o. This study revealed the unique molecular architecture of the hybrid complex from *C*.* glutamicum* and the compact‐sized complex would provide an advantage to determine the whole structure of the unusual hybrid complex.

## Graphical abstract

This study shows the unique molecular architecture of the PDH‐ODH hybrid complex in *Corynebacterium glutamicum*, in which both *Cg*E1p and *Cg*E1o associate with the *Cg*E2‐E3 subcomplex. The core‐forming *Cg*E2‐E3 probably consists of six copies of *Cg*E2, which is relatively compact compared with PDH and ODH in other microorganisms that have 24 copies of E2. The fragility of the *Cg*PDH complex and the unusual multimerization of *Cg*E1o are also presented.

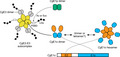

## 1 INTRODUCTION

Pyruvate dehydrogenase (PDH) and 2‐oxoglutarate dehydrogenase (ODH) are critical enzymes in central carbon metabolism and generate vital high energy compounds acetyl‐CoA and succinyl‐CoA, respectively; both of which are major CoA derivatives in cell and serve as substrates for protein acetylation and succinylation (Komine‐Abe et al., 2017; Mizuno et al., 2016; Nagano‐Shoji et al., 2017). PDH and ODH are large enzyme complexes (∼10 MDa) composed of three subunits; E1, E2, and E3. E1 (EC 1.4.2.1) is involved in thiamine pyrophosphate‐dependent oxidative decarboxylation of 2‐oxoacid (pyruvate or 2‐oxoglutarate) with the concomitant transfer of the corresponding acyl group to a lipoyl group attached on a lysine residue of E2. E2 (EC 2.3.1.12) is a dihydrolipoyl acyltransferase that catalyzes the transfer of the acyl group to CoA. E3 (EC 1.8.1.4) is a dihydrolipoyl dehydrogenase that is responsible for FAD‐dependent reoxidation of dihydrolipoamide to lipoamide on E2 to generate NADH (Perham, 2000; Patel, Nemeria, Furey, & Jordan, 2014). The complexes are organized in both structural and functional aspects using E2 as the core. E2 protein consists of three different types of domains: one to three N‐terminal lipoyl domains (~80 amino acids each), a peripheral subunit‐binding domain (PSBD, ~45 amino acids), and a core‐forming C‐terminal catalytic acyltransferase domain (~250 amino acids). As demonstrated by the crystal structures of PDH and ODH from several organisms (Izard et al., 1999; Knapp et al., 1998; Mattevi et al., 1992, 1993; Wang et al., 2014), E2 trimer is the principal building block to be assembled into an octahedral (24‐mer) or icosahedral (60‐mer) symmetrical shape to form the inner core. Both E1 and E3 exist as a dimer and are noncovalently tethered to the PSBD of E2. In many organisms, including *Escherichia coli*, the E3 protein is shared by PDH and ODH, while specific E1 and E2 proteins are utilized for each enzyme complex.

PDH and ODH from the order Corynebacteriales including *Corynebacterium glutamicum* have several unique features. First, there is only a single gene encoding E2 in the genome, and the same E2, as well as E3, proteins are used by PDH and ODH. Second, there exists a variant protein of E1o with an extra E2 acyltransferase domain in the N‐terminal region, which corresponds to OdhA (*Cg*E1o, NCgl1084) in *C*. *glutamicum* (Usuda et al., 1996) and Kgd or SucA in *Mycobacterium* (Tian et al. 2005; Wagner et al. 2019). The deletion of *odhA* resulted in the loss of ODH activity, and OdhA certainly functions as E1o (Hoffelder et al. 2010). *Mycobacterium* SucA functions as a multifunctional enzyme capable of reductive succinyl‐transfer to a lipoyl residue via the canonical dehydrogenase reaction (Wagner et al. 2011), nonreductive decarboxylation of α‐ketoglutarate (2‐oxoglutarate) to produce succinic semialdehyde (Tian et al., 2005; Wagner et al., 2011), and carboligation with glyoxylate to give 2‐hydroxy‐3‐oxoadipate (de Carvalho et al., 2010; Wagner et al., 2011). Third, a small regulatory protein (OdhI in *C*. *glutamicum* and GarA in *Mycobacterium*) interacts with the E1o variant to control the enzymatic activity (Niebisch et al., 2006; Nott et al., 2009). OdhI is composed of two domains: an N‐terminal domain, containing phosphorylation sites, and a C‐terminal forkhead‐associated (FHA) domain, recognizing phosphorylated serine/threonine. The FHA domain also establishes the binding to *Cg*E1o (Krawczyk et al., 2010). When the relevant N‐terminal sites are phosphorylated, OdhI undergoes a conformational change through the interaction of the FHA domain with the phosphorylated sites, releasing ODH from the inhibition by OdhI (Barthe et al., 2009).

In a previous study that investigated the subunit organization of *C*. *glutamicum*, ODH, *Cg*E2 (AceF, NCgl2126), and *Cg*E3 (Lpd, NCgl0355) were copurified along with a tagged *Cg*E1o as expected. Interestingly, *Cg*E1p (AceE, NCgl2167) was also copurified with the ODH complex (Niebisch et al., 2006). Furthermore, when a tagged *Cg*E1p was used as prey, all the components of the ODH complex were copurified (Niebisch et al., 2006). This was the first evidence to suggest the presence of a PDH‐ODH hybrid complex in *C*. *glutamicum*. Further enzymatic and genetic studies revealed that each E2 acyltransferase domain of *Cg*E2 and *Cg*E1o specifically catalyzes the transacetylase and transsuccinylase reaction, respectively, suggesting that the E2 catalytic domain of *Cg*E2 and *Cg*E1o is responsible for PDH and ODH activities (Hoffelder et al., 2010). It was also revealed that *Cg*E1o and also *Cg*E2 are required for the ODH activity (Hoffelder et al., 2010). Recent structural studies of *Mycobacterium smegmatis* SucA, which is the homolog of *Cg*E1o, revealed that the E1o catalytic domain is involved in generating a ThDP‐bound decarboxylation intermediate derived from 2‐oxoglutarate. This decarboxylation intermediate undergoes conformational changes from the α‐carbanion to the enamine form and is reductively transferred to a lipoyl residue on E2 (Wagner et al., 2014; Wagner et al., 2019). All the pieces of evidence strongly support the presence of the PDH‐ODH hybrid complex. However, the molecular architecture, including the subunit stoichiometry and arrangement of the hybrid complex, remains to be elucidated. In the present study, we aimed to characterize the *C*.* glutamicum* PDH‐ODH complex *in vivo* and *in vitro* and show the unique molecular architecture of the hybrid complex.

## 2 MATERIALS AND METHODS

### 2.1 Bacterial strains and culture conditions

*Corynebacterium glutamicum* ATCC13869 was used as the wild‐type strain. All strains and plasmids used in this study are shown in Table 1. *Corynebacterium glutamicum* cells were grown in 25 ml of glutamate production medium (Mizuno et al., 2016) at 31.5°C in 500‐ml baffled flasks on a rotary shaker at 100 rpm (70 mm of the shaking width, Takasaki Kagaku, Japan). Tween 40 (1.5 g/l) was added after 3 h of cultivation to induce l‐glutamate production, and the same volume of distilled water was added for control (uninduced). Nine‐hour cultivated cells were used for the lysate preparation.

**TABLE 1:** Bacterial strains and plasmids used in this study

	Description	Source or reference
*Corynebacterium glutamicum* strain		
ATCC13869	Wild‐type strain	Laboratory stock
Plasmids		
pAA62	pET21a expressing C‐terminal His‐tagged *Cg*E1o (OdhA)	Komine‐Abe et al. (2017)
pAA77	pET21a expressing N‐terminal Strep‐tagged OdhI	Komine‐Abe et al. (2017)
pKH4	pET21a expressing C‐terminal His‐tagged *Cg*E1p (AceE)	This study
pKH13	pRSFDuet expressing N‐terminal His‐ and Flag‐tagged *Cg*E3 (Lpd) and *Cg*E2 (AceF)	This study

### 2.2 Ultracentrifuge analysis

*Corynebacterium glutamicum* cells were lysed in TESG15 buffer (50 mM TES‐NaOH [pH 7.6] and 15% glycerol) containing 1 mM PMSF, 1 mM DTT, 10 µg/ml DNase, and 10 µg/ml RNase at high pressure using EmulsiFlex‐B15 (Avestin Inc., Ottawa). After cell debris removal by centrifugation (7000 *g*, 10 min, 4°C), the cleared lysate containing 1.2 mg of protein was layered onto a 10%–30% or 15%–45% sucrose density gradient in 50 mM TES‐NaOH (pH 7.6). The gradient was centrifuged at 36,000 rpm (max. 230,000 *g*) in a HITACHI P40ST rotor for 17 h at 4°C. After centrifugation, the gradient was fractionated using a Piston Gradient Fractionator (BioComP Instruments, Fredericton) into 21 fractions of 0.5 ml and the bottom fraction. *Escherichia coli* DH5α cells were grown in 2 × YT medium until the OD_660_ reached 3.5 and processed using the same ultracentrifuge protocol. Aliquots (10 μl) were separated by SDS‐PAGE and subjected to Western blot analysis to detect each subunit of PDH and ODH. Denatured samples were prepared by boiling for 5 min in the presence of 1% SDS before ultracentrifugation.

### 2.3 Western blot analysis

Western blot analysis was performed as described previously (Mizuno et al., 2016). The rabbit polyclonal anti‐*Cg*AceE and anti‐*Cg*AceF antibodies were raised against the commercially synthesized NCgl2167 (904‐KFKLDDPTSVSVDPNAPEE‐922) and NCgl2126 (1‐MAFSVEMPELGESVT‐15) peptides, respectively (Sigma‐Genosys LP, Texas). Primary antibodies used are as follows: rabbit polyclonal anti‐*Cg*AceE (1:10,000); rabbit polyclonal anti‐*Cg*AceF (1:5000); rabbit polyclonal anti‐*Cg*OdhA (1:10,000) (Kim et al., 2011); rabbit polyclonal anti‐*Ec*AceE (1:10,000); rabbit polyclonal anti‐*Ec*AceF (1:5000); rabbit polyclonal anti‐*Ec*SucA (1:10,000); rabbit polyclonal anti‐*Ec*SucB (1:10,000); rabbit polyclonal anti‐*Ec*Lpd (1:10,000) (provided by Shimada and Tanaka); rabbit polyclonal anti‐RplC from *Bacillus subtilis* (1:10,000) (provided by Kawamura); and rabbit anti‐lipoic acid antibody (Calbiochem, 437695) (1:2000).

### 2.4 Preparation of recombinant proteins

Recombinant *Cg*E1o protein was expressed as a C‐terminal His‐tagged protein using the pET‐21a vector (pAA62) (Komine‐Abe et al., 2017). Recombinant *Cg*E1p protein was expressed as a C‐terminal His‐ and Flag‐tagged protein using the pET‐21a vector (pKH4). Recombinant *Cg*E2 and *Cg*E3 proteins were co‐expressed using the pRSFDuet vector (pKH13). N‐terminal His‐ and Flag‐tagged *Cg*E3 enabled copurification of *Cg*E2 and *Cg*E3 as a *Cg*E2‐E3 subcomplex. Recombinant OdhI protein was expressed as an N‐terminal Strep‐tagged protein (pAA77) (Komine‐Abe et al., 2017).

All recombinant proteins were produced in *Escherichia coli* BL21‐CodonPlus (DE3) cells. *Escherichia coli* cells were grown in 2×YT medium at 37°C for 3 h and then cultivated at 25°C for 18 h in the presence of 0.5 mM IPTG to induce enzyme expression. The cells were harvested and lysed in TESG10N (100 mM TES‐NaOH [pH 7.6], 150 mM NaCl, and 10% [w/v] glycerol) containing 1 mM PMSF, 1 mM DTT, 10 µg/ml DNase, and 10 µg/ml RNase, followed by affinity purification using Ni‐NTA His Bind resin (Merck Millipore, Burlington). His‐tagged proteins were eluted with TESG10N containing 200 mM imidazole. The eluted proteins were dialyzed against the TESG10N buffer.

### 2.5 In vitro reconstitution of PDH and ODH

Dialyzed or gel filtration purified *Cg*E2‐E3 proteins were mixed with *Cg*E1p and/or *Cg*E1o in TESG10N buffer and incubated on ice for 30 min. Then, the sample was used for enzyme assays or gel filtration chromatography.

### 2.6 Gel filtration chromatography

Gel filtration chromatography was performed using a HiLoad 26/600 Superdex 200 pg column (GE Healthcare, Chicago) or a Superose 6 10/300 GL column (GE Healthcare, Chicago) with TESG10N buffer. For a Superdex 200 column, 2 or 5 ml of sample was applied. Chromatography was performed at a flow rate of 1.0 ml per min for 600 min, and 5 ml fractions were collected. For a Superose 6 column, 0.2 ml of sample was applied. Chromatography was performed at a flow rate of 0.2 ml per min for 250 min, and 0.5 ml fractions were collected.

### 2.7 Enzyme assays

PDH and ODH activities were assayed as described previously (Komine‐Abe et al., 2017). Recombinant *Cg*E2‐E3 and *Cg*E1 proteins were mixed and kept on ice for 30 min. Aliquots (20–50 μl) of the mixture were incubated in a reaction mixture (1 ml) containing 100 mM TES‐NaOH (pH 7.6), 0.2 mM coenzyme A, 0.3 mM thiamine pyrophosphate, 3 mM L‐cysteine, 5 mM MgCl_2_, 1 mM oxidized form of 3‐acetylpyridine adenine dinucleotide (APAD^+^; Oriental Yeast Co., Ltd., Japan), and 6 mM pyruvate (for PDH) or 1 mM 2‐oxoglutarate (for ODH). Assays were performed at 30°C, and the initial rate of APAD^+^ reduction was monitored using spectrophotometry at 365 nm. Endogenous APAD^+^ reduction in the absence of the substrates was subtracted from the APAD^+^ reduction in the presence of the substrates. One unit of activity was defined as the reduction of 1 nmol of APAD^+^ (*ε* = 9.1 l μmol^−1 ^cm^−1^) per min.

## 3 RESULTS

### 3.1 Detection of the PDH‐ODH hybrid complex in *C. glutamicum* lysates using ultracentrifuge analysis

**FIGURE 1:**
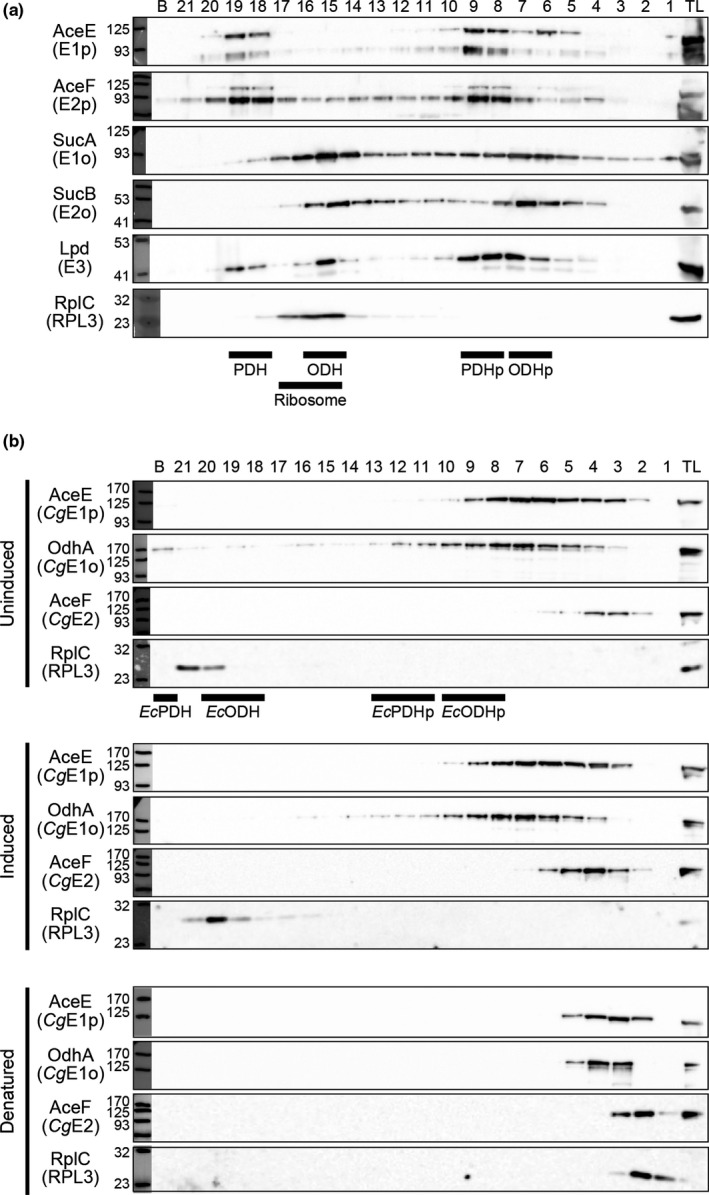
Detection of PDH and ODH *in vivo*. (a) Ultracentrifuge analysis of *Escherichia coli* lysates. The total lysate (TL) was sedimented using a 15%–45% sucrose density gradient. Aliquots (10 μl) of 21‐fractionated and the bottom (b) samples were separated using 8% or 10% SDS‐PAGE, followed by Western blot analysis to detect each subunit. Black bars indicate the positions of PDH, ODH, and ribosomes detected. PDH_p_ and ODH_p_ represent partial complexes of PDH and ODH, respectively. A representative data set of two biologically independent experiments is shown. (b) Ultracentrifuge analysis of *Corynebacterium glutamicum* lysates. The TL was sedimented using a 10%–30% sucrose density gradient. Uninduced, conditions without Tween 40 as an inducer; induced, Tween 40‐induced glutamate‐producing conditions; denatured, denatured samples of uninduced conditions. Detection of each subunit was performed as shown in (a). Black bars indicate the positions of *Escherichia coli* PDH, ODH, and ribosomes detected in the same conditions (see Figure A1). A representative data set of three biologically independent experiments is shown

To investigate the molecular architectures of PDH and ODH in *C*.* glutamicum* lysates, we performed ultracentrifuge analysis using a sucrose density gradient. We first analyzed *E*.* coli* lysate samples, since the molecular architectures of the PDH and ODH complexes in *E*.* coli* are well characterized. Previous ultracentrifuge analysis showed that the *E*.* coli* PDH and ODH are large complexes with the molecular mass of 4.8 MDa and 2.4 MDa, respectively (Koike et al., 1960). Structural studies revealed that in both complexes, 24 copies of E2 subunit form an octahedral inner core, and up to 24 dimers of either E1 or E3 associate with the E2 core (Knapp et al., 1998; Murphy & Jensen, 2005; Reed et al., 1975). In our ultracentrifuge analysis of *E*.* coli* lysates using a 15%–45% sucrose density gradient (Figure 1a), the PDH subunit proteins AceE (E1p), AceF (E2p), and Lpd (E3) were detected in fractions 18‐19, representing a PDH complex. The three PDH subunits were also detected in fractions 8‐9. Previous studies reported that the PDH complex was present in multiple forms, a major 56‐60 S component (a full complex) as well as minor 86‐90 S and 20‐26 S (a partial complex) components (Danson et al., 1979; Koike et al., 1960). The PDH complexes in fractions 18‐19 and 8‐9 were assigned as the full and partial complexes of PDH, respectively. Meanwhile, SucA (E1o), SucB (E2o), and Lpd (E3) of the ODH complex were detected in fractions 15‐16 and 6‐7, which were likely the full and partial complexes of ODH, corresponding to major 37 S and minor 22 S components, according to a previous sedimentation analysis (Koike et al., 1960), respectively. We also detected the ribosomal large subunit protein L3 (RplC) in fractions 15‐17, representing ribosomes (an approx. molecular mass of 2.7 MDa). Thus, our centrifugation system could assign PDH, ODH, and ribosomes in fractions with the expected molecular sizes and confirmed that PDH and ODH are different complexes in *E*.* coli*. These results assured that our ultracentrifuge system was reliable to analyze the molecular architecture of large protein complexes like PDH and ODH.

**FIGURE A1:**
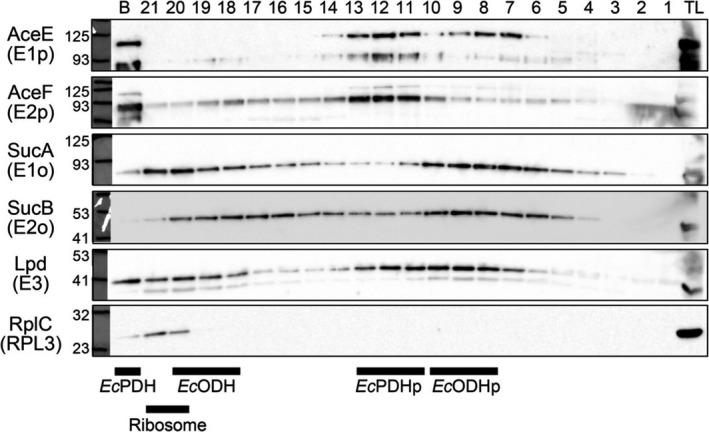
Ultracentrifuge analysis of *Escherichia coli* lysates using a 10%–30% sucrose density gradient. The total lysates (TL) of *Escherichia coli* were processed using the same ultracentrifuge protocol as Figure 1b. Aliquots (10 μl) of 21‐fractionated and the bottom (b) samples were separated using 8% or 10% SDS‐PAGE, followed by western blot analysis to detect each subunit. Black bars indicate the positions of *Ec*PDH, *Ec*ODH and ribosome detected. *Ec*PDHp and *Ec*ODHp represent partial complexes of *Ec*PDH and *Ec*ODH, respectively. The positions of *Ec*PDH, *Ec*ODH, *Ec*PDHp, and *Ec*ODHp are indicated in Figure 1b

We next analyzed *C*.* glutamicum* lysate samples prepared from cells grown in glutamate production medium without Tween 40 (as an inducer) using ultracentrifugation in a 10%–30% sucrose density gradient. *Cg*E1p, *Cg*E1o, and *Cg*E2 proteins were detected in the “light” fraction 4 (and also 5‐6) (Figure 1b). *Cg*E2 was migrated to the lighter fractions (2 and 3) in SDS‐treated denatured samples. We thus speculated that *Cg*E1p, *Cg*E1o, and *Cg*E2 formed a PDH‐ODH hybrid complex which was eluted in fraction 4, although the fractions containing *Cg*E3 could not be determined due to the unavailability of the specific antibody. The molecular mass of a hybrid PDH‐ODH complex could be much smaller than that of the *E*.* coli* PDH or ODH complex (Figure 1b, Figure A1). Interestingly, *Cg*E1p and *Cg*E1o migrated to higher molecular mass fractions (fraction 7 and more) which did not contain *Cg*E2. This may suggest that the two E1 proteins interact with unknown proteins or are multimerized.

We also performed a similar analysis using lysates from cells grown in Tween 40‐triggered glutamate‐producing conditions and compared the sedimentation patterns of the PDH and ODH subunit proteins. The *Cg*E2 was constantly detected in higher molecular mass fractions in glutamate‐producing conditions compared with nonproducing conditions (Figure 1b).

### 3.2 Reconstitution of the PDH and ODH activities in vitro

**FIGURE 2:**
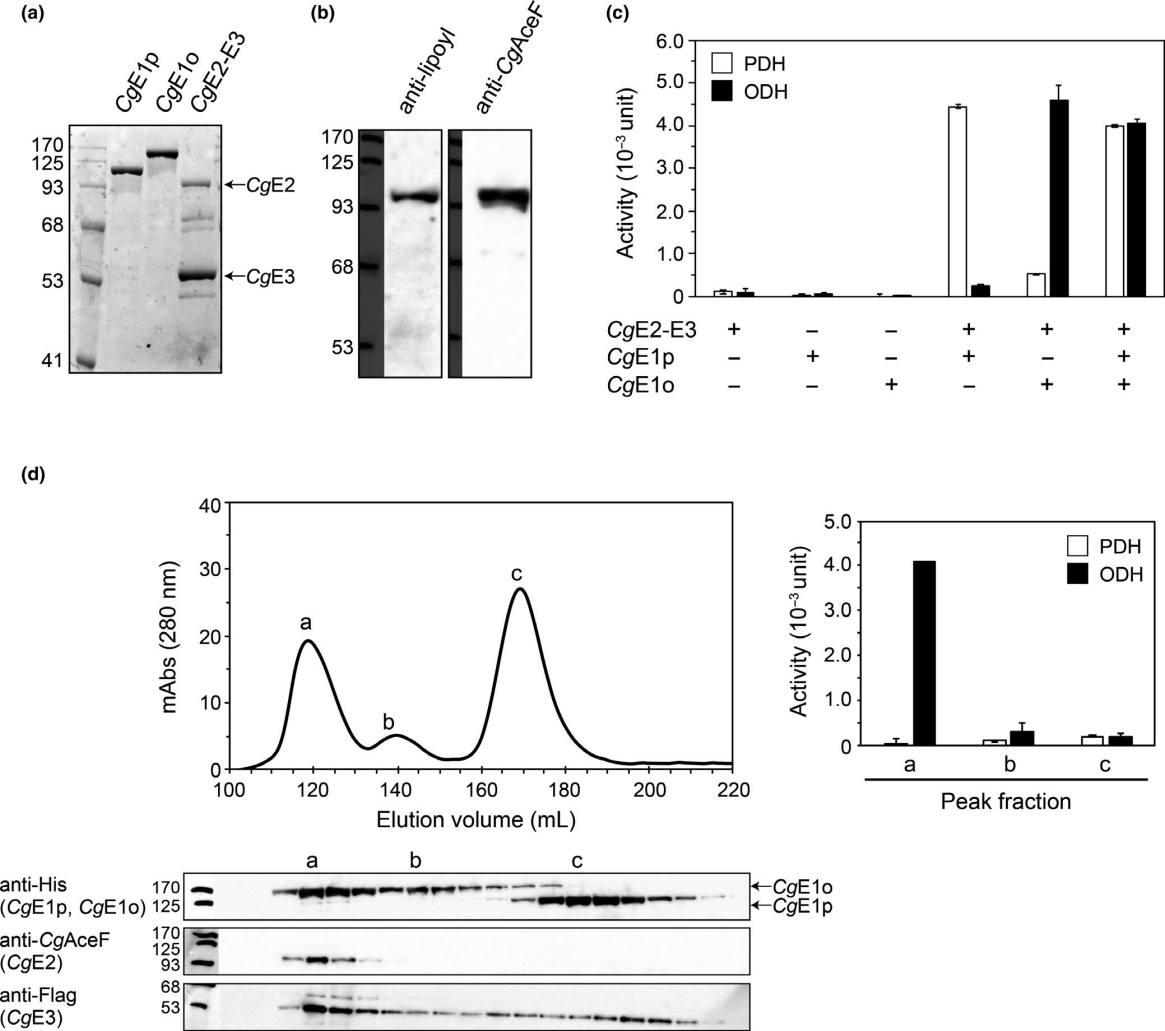
*In vitro* reconstitution of PDH and ODH activities. (a) Affinity purification of His‐tagged *Cg*E1p, *Cg*E1o, and *Cg*E2‐E3 of *Corynebacterium glutamicum*. Affinity‐purified samples (1.5 μg) were separated using 10% SDS‐PAGE and detected using CBB staining. (b) Detection of lipoyl group attachment on *Cg*E2. Affinity‐purified *Cg*E2 (2 μg), separated using 10% SDS‐PAGE, was detected using Western blot analysis with anti‐lipoic acid (left) and anti‐*Cg*E2 (right) antibodies. (c) *Cg*E1p‐dependent PDH and *Cg*E1o‐dependent ODH activities. Affinity‐purified *Cg*E2‐E3 (110 μg) was mixed with *Cg*E1p (70 μg), *Cg*E1o (90 μg), or both and subjected to PDH (white columns) and ODH (black columns) activity assays. Data are shown as the mean and standard deviation of triplicate assays. A representative data set of three independent experiments is shown. (d) Gel filtration chromatogram of the reconstituted sample including *Cg*E1p, *Cg*E1o, and *Cg*E2‐E3 using a Superdex 200 column (upper). Aliquots (5 μl) of fractionated samples (5 ml each) were analyzed using Western blotting to detect each subunit (lower). Samples of three peak fractions (a, b, and c) were subjected to PDH and ODH activity assays (right). Data are shown as the mean and standard deviation of triplicate assays

**FIGURE A2:**
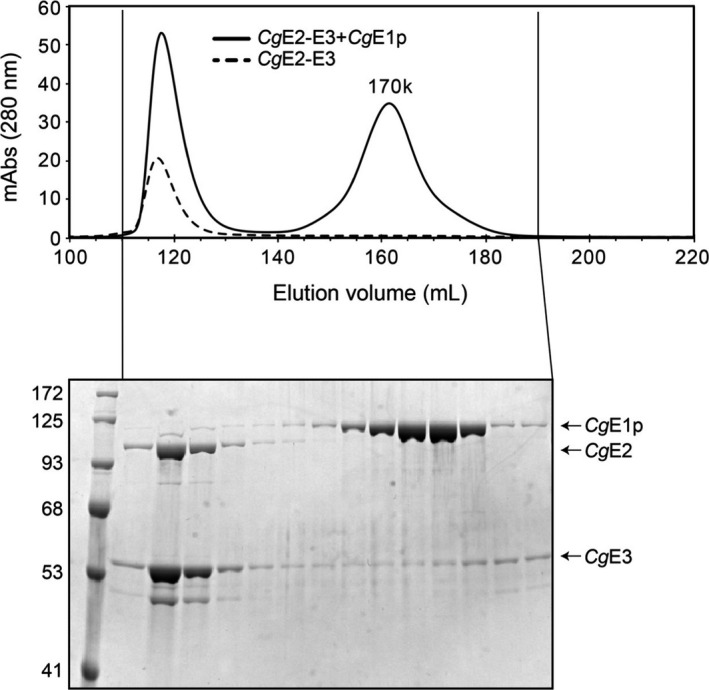
Gel filtration chromatography of a mixture sample of the *Cg*E1p and the *Cg*E2‐E3 subcomplex. Chromatograms of the mixture sample (solid line) and individual *Cg*E2‐E3 (broken line) using a Superdex 200 column are shown (upper). Aliquots (5 μl) of fractions (5 ml each) were analyzed using 10% SDS‐PAGE and the CBB staining gel image is shown (lower). An estimated molecular mass of the peak fraction containing *Cg*E1p was 170 kDa

We tried to reconstitute PDH and ODH activities *in vitro* using subunit proteins prepared from *E*. *coli*. We successfully copurified *Cg*E2 with *Cg*E3 using the N‐terminal His‐tagging of *Cg*E3 (Figure 2a). The interaction of *Cg*E2 with *Cg*E3 was retained in gel filtration chromatography (Figure 2d, Figure A2), suggesting the formation of an E2‐E3 subcomplex rather than a mixture of the two proteins. The recombinant *Cg*E2 contained an attached lipoyl group, as detected using Western blot analysis with an anti‐lipoyl antibody (Figure 2b). Incubation of the affinity‐purified *Cg*E2‐E3 with *Cg*E1p and *Cg*E1o resulted in PDH and ODH activities, respectively (Figure 2c). When both *Cg*E1p and *Cg*E1o were added to a reaction mixture, PDH and ODH activities were detected. These results indicated that PDH and ODH activities were successfully reconstituted *in vitro*.

Next, we analyzed the reconstituted sample containing *Cg*E1o, *Cg*E1p, and *Cg*E2‐E3 using gel filtration chromatography. Three peak fractions (*a*,* b*, and *c*) were observed in the chromatogram. Western blot analysis using specific antibodies revealed that the *a* fraction contained *Cg*E1o, *Cg*E2, and *Cg*E3 proteins, the *b* fraction contained *Cg*E1o and *Cg*E3, and the *c* fraction majorly contained *Cg*E1p (Figure 2d, left). ODH activity was detected in the *a* fraction, indicating that the peak *a* represents the ODH complex, in which *Cg*E1o was associated with the *Cg*E2‐E3 subcomplex. On the other hand, no PDH activity was detected in any peak fraction (Figure 2d, right). Also, when *Cg*E2‐E3 was incubated only with *Cg*E1p, *Cg*E2‐E3 and *Cg*E1p were eluted in separate fractions (Figure A2). These results suggested that the association of *Cg*E1p with *Cg*E2‐E3 was too weak to be retained during gel filtration.

### 3.3 Subunit stoichiometry of the CgE2‐E3 subcomplex and ODH complex

**FIGURE 3:**
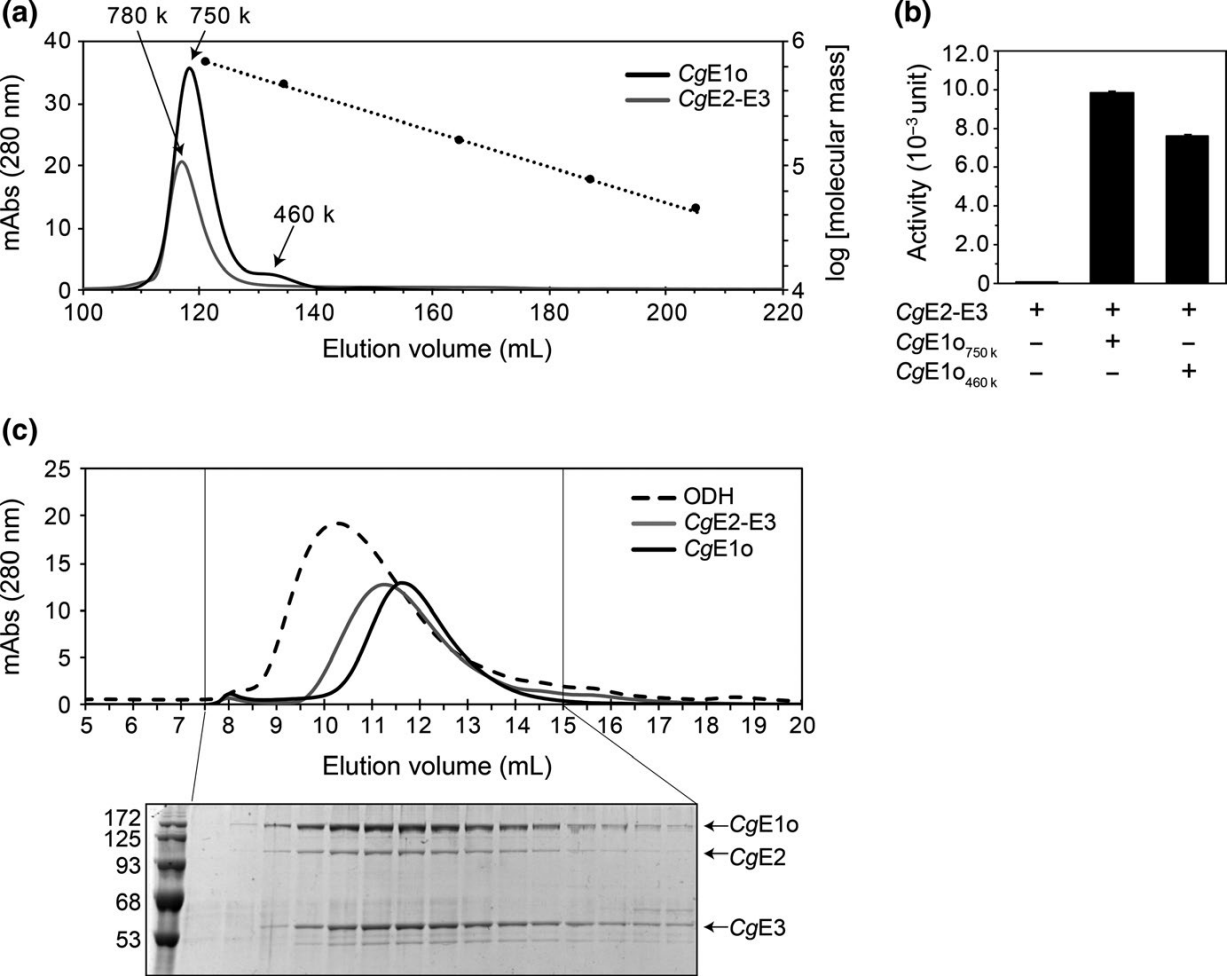
Estimation of molecular masses of the *Cg*E1o component, the *Cg*E2‐E3 subcomplex, and ODH complex. (a) Gel filtration chromatograms of the E1o (black line) and the E2‐E3 subcomplex (gray line) using a Superdex 200 column. The dotted line indicates a standard curve of molecular weight standards (thyroglobulin, 669 kDa; ferritin, 440 kDa; aldolase, 158 kDa; conalbumin, 75 kDa; and ovalbumin, 44 kDa). A representative data set of two independent experiments is shown. (b) Both *Cg*E1o fractions of 750 kDa (*Cg*E1o_750k_) and 460 kDa (*Cg*E1o_460k_) provided ODH activity. *Cg*E1o_750k_ or *Cg*E1o_460k_ (20 μg) was mixed with E2‐E3 (15 μg) and subjected to enzyme assays. Data are shown as the mean and standard deviation of triplicate assays. (c) Gel filtration chromatograms of the ODH complex (a mixture of E1o with *Cg*E2‐E3, broken line) using a Superose 6 column (upper). Chromatograms of individual *Cg*E1o (black line) and *Cg*E2‐E3 (gray line) are also shown. Aliquots (10 μl) of fractions (0.5 ml each) indicated were analyzed using 10% SDS‐PAGE, and the CBB staining gel image is also shown (lower)

**FIGURE A3:**
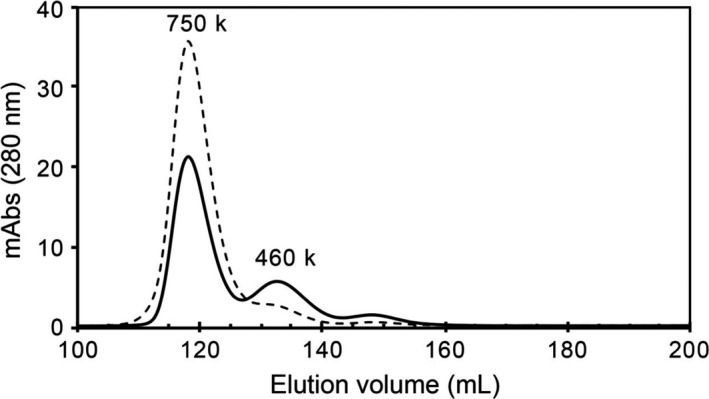
Gel filtration chromatography of the *Cg*E1o460k fraction. The fraction containing *Cg*E1o460k were collected and subjected to gel filtration chromatography, which provided two peaks of 750 kDa and 460 kDa again. The chromatography pattern of *Cg*E1o in Figure 3a is shown with a broken line as a reference

The subunit stoichiometry of *Cg*E2‐E3 and ODH complexes was analyzed using gel filtration chromatography with molecular size standards. Although the *Cg*E2‐E3 subcomplex was eluted outside the range of the size standards, the molecular mass of the subcomplex was estimated to be 780 kDa (Figure 3a). *Cg*E2 and *Cg*E3 proteins show 47% and 53% similarity with *E*. *coli* E2p and E3, respectively, and following relevant estimations were done according to previous research (Chandrasekhar et al., 2013; Wang et al., 2014). The subunit stoichiometry of the *Cg*E2‐E3 subcomplex was estimated to be six copies of *Cg*E2 (theoretical molecular mass of 70,905 Da) and eight copies of *Cg*E3 (theoretical molecular mass of 50,652 Da), giving two E2 trimers and four E3 dimers (theoretically 831 kDa in total). *Cg*E1o (theoretical molecular mass of 134,664 Da) was eluted in two separated peak fractions, corresponding to 750 kDa and 460 kDa for major and minor peaks, respectively (Figure 3a). The 750 kDa and 460 kDa multimers of *Cg*E1o were estimated to be a hexamer (theoretically 808 kDa), and a tetramer (theoretically 539 kDa) or a trimer (theoretically 404 kDa). The fraction of 460 kDa (*Cg*E1o_460k_) was collected and subjected to gel filtration chromatography, which provided two peaks of 750 kDa and 460 kDa again (Figure A3). Since both *Cg*E1o‐containing fractions exhibited ODH activity when incubated with *Cg*E2‐E3, they were not inactive aggregates but functional multimers (Figure 3b). Therefore, we assumed that *Cg*E1o is present at equilibrium between the two multimer forms in solution.

Next, we tried to determine the molecular mass of the ODH complex. The mixture sample containing *Cg*E1o and *Cg*E2‐E3 provided a new peak fraction with a higher molecular mass than the individual *Cg*E1o and *Cg*E2‐E3 samples (Figure 3c). Since the new peak fraction contained *Cg*E1o, *Cg*E2, and *Cg*E3 (Figure 3c) and exhibited ODH activity, the active ODH complex was indeed reconstituted *in vitro*. The exact molecular mass could not be estimated for the ODH complex because it ranged out of the molecular size standards. However, it was estimated to be approximately 940 kDa.

As described above, the full PDH complex, consisting of *Cg*E1p, *Cg*E2, and *Cg*E3, was not detected in our gel filtration chromatography. The *Cg*E1p (a theoretical molecular mass of 102,826 Da) was mainly eluted in a peak fraction with the estimated molecular size of 170 kDa, which likely represents a dimeric form (theoretically 206 kDa) in solution (Figure A2).

### 3.4 Kinetic analysis of in vitro reconstituted PDH and ODH

**FIGURE 4:**
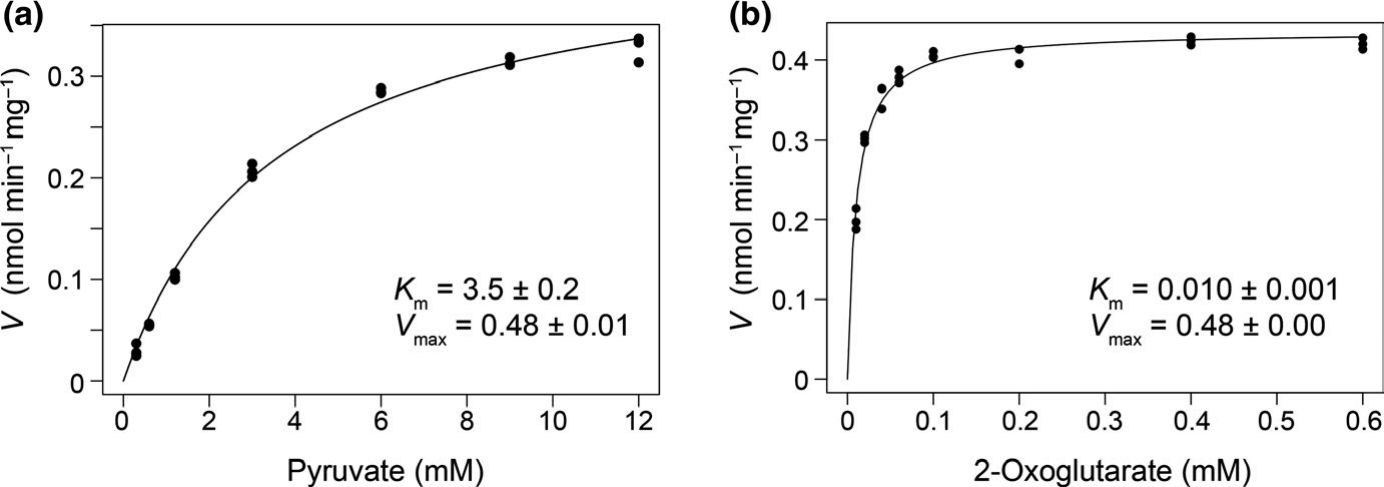
Kinetic analysis of PDH (a) and ODH (b) activities. A mixture of *Cg*E1p (15 μg) or *Cg*E1o (20 μg) with *Cg*E2‐E3 (15 μg) was incubated on ice for 30 min for reconstitution and then subjected to enzyme assays. Dots show individual data of triplicate assays

**FIGURE A4:**
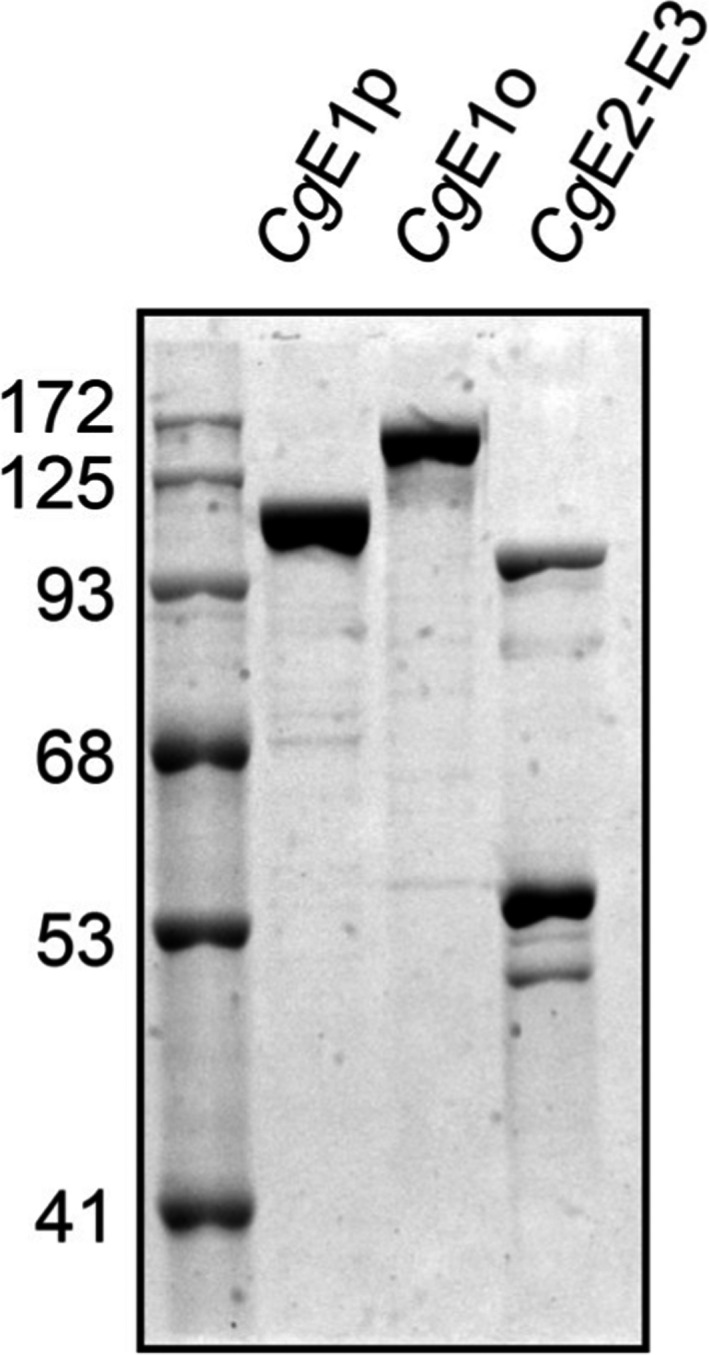
The purity of gel‐filtration purified His‐tagged *Cg*E1p, *Cg*E1o, and *Cg*E2‐ E3 proteins. Purified samples (2 μg) were separated using 10% SDS‐PAGE and detected using CBB staining. These protein samples were used for kinetic analysis (Figure 4)

We performed kinetic analyses of the reconstituted PDH and ODH complexes. For this analysis, the affinity‐purified proteins were further purified using gel filtration chromatography to remove contaminated proteins (Figure A4). As a result of the enzyme assays using a mixture of *Cg*E2‐E3 and *Cg*E1p or *Cg*E1o, shown in Figure 4, the apparent *K*_m_ value of the reconstituted PDH complex for pyruvate was 3.5 ± 0.2 mM with a *V*_max_ of 0.48 ± 0.01 nmol min^−1 ^mg^−1^, and the apparent *K*_m_ of the reconstituted ODH complex for 2‐oxoglutarate was 0.010 ± 0.001 mM with a *V*_max_ of 0.48 ± 0.00 nmol min^−1 ^mg^−1^. Our apparent *K*_m_ value of the PDH complex for pyruvate was comparable to that reported previously using *C*.* glutamicum* lysates (1.7 mM), while our apparent *K*_m_ of the ODH complex for 2‐oxoglutarate was one order smaller than that determined using lysates (0.13 mM) (Hoffelder et al., 2010).

### 3.5 CgE1p‐ and CgE1o‐dependent inhibition of ODH and PDH, respectively

**FIGURE 5:**
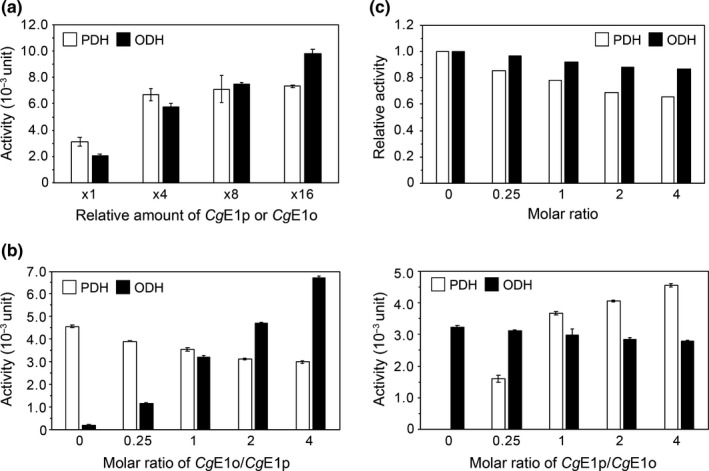
*Cg*E1p‐dependent inhibition of ODH and *Cg*E1o‐dependent inhibition of PDH. (a) Dose dependency of PDH and ODH activities. Various amounts (37, 150, 300, and 600 pmol) of *Cg*E1p or *Cg*E1o were mixed with a fixed amount of *Cg*E2‐E3 (15 μg, 18 pmol) and subjected to enzyme assays. The *Cg*E1p (3.8 μg, 37 pmol) and *Cg*E1o (5.0 μg, 37 pmol) are defined as one amount. Data are shown as the mean and standard deviation of triplicate assays. (b) Competitive inhibition of PDH and ODH activities. PDH and ODH complexes were reconstituted by mixing *Cg*E1p (15 μg, 150 pmol) and *Cg*E1o (20 μg, 150 pmol) with *Cg*E2‐E3 (15 μg, 18 pmol), separately; 37, 150, 300, and 600 pmol of *Cg*E1o were added to the PDH complex (left), and 37, 150, 300, and 600 pmol of *Cg*E1p were added to the ODH complex (right) and subjected to enzyme assays. The molar ratio of E1p and E1o is shown on the *X*‐axis. Data are shown as the mean and standard deviation of triplicate assays. A representative data set of two independent experiments is shown. (c) *Cg*E1o‐dependent PDH inhibition (white columns) and *Cg*E1p‐dependent ODH inhibition (black columns). The data were originated from Figure 5b and show the relative activities compared to the ones without the other E1 subunit

*Cg*E1p and *Cg*E1o subunits associate with *Cg*E2‐E3 in a complex. *Cg*E1p (or *Cg*E1o) may compete with *Cg*E1o (or *Cg*E1p) for binding to *Cg*E2‐E3, which results in competitive inhibition of ODH and PDH via *Cg*E1p and *Cg*E1o, respectively. We examined the effect of *Cg*E1o (or *Cg*E1p) addition on PDH or ODH activity. We first examined the dose dependency of each *Cg*E1 on the activity of the cognate enzyme. PDH activity was saturated when 15 μg (150 pmol) of *Cg*E1p was added to a fixed amount (15 μg, 18 pmol) of *Cg*E2‐E3 (Figure 5a). Meanwhile, ODH activity was not saturated even with 80 μg (600 pmol) of *Cg*E1o in the reaction mixture containing the same amount of *Cg*E2‐E3. Next, we added an increased amount of *Cg*E1o to the above reconstituted PDH mixture. As the molar ratio of *Cg*E1o to *Cg*E1p increased, PDH activity decreased (Figure 5b, left, white columns). Similarly, ODH activity decreased as the molar ratio of *Cg*E1p to *Cg*E1o increased (Figure 5b, right, black columns). The inhibitory effect of *Cg*E1o on PDH activity was stronger than that of *Cg*E1p on ODH (Figure 5c). These results suggested that *Cg*E1p and *Cg*E1o competed with each other for *Cg*E2‐E3 to form the PDH‐ODH hybrid complex.

### 3.6 The effect of OdhI

**FIGURE 6:**
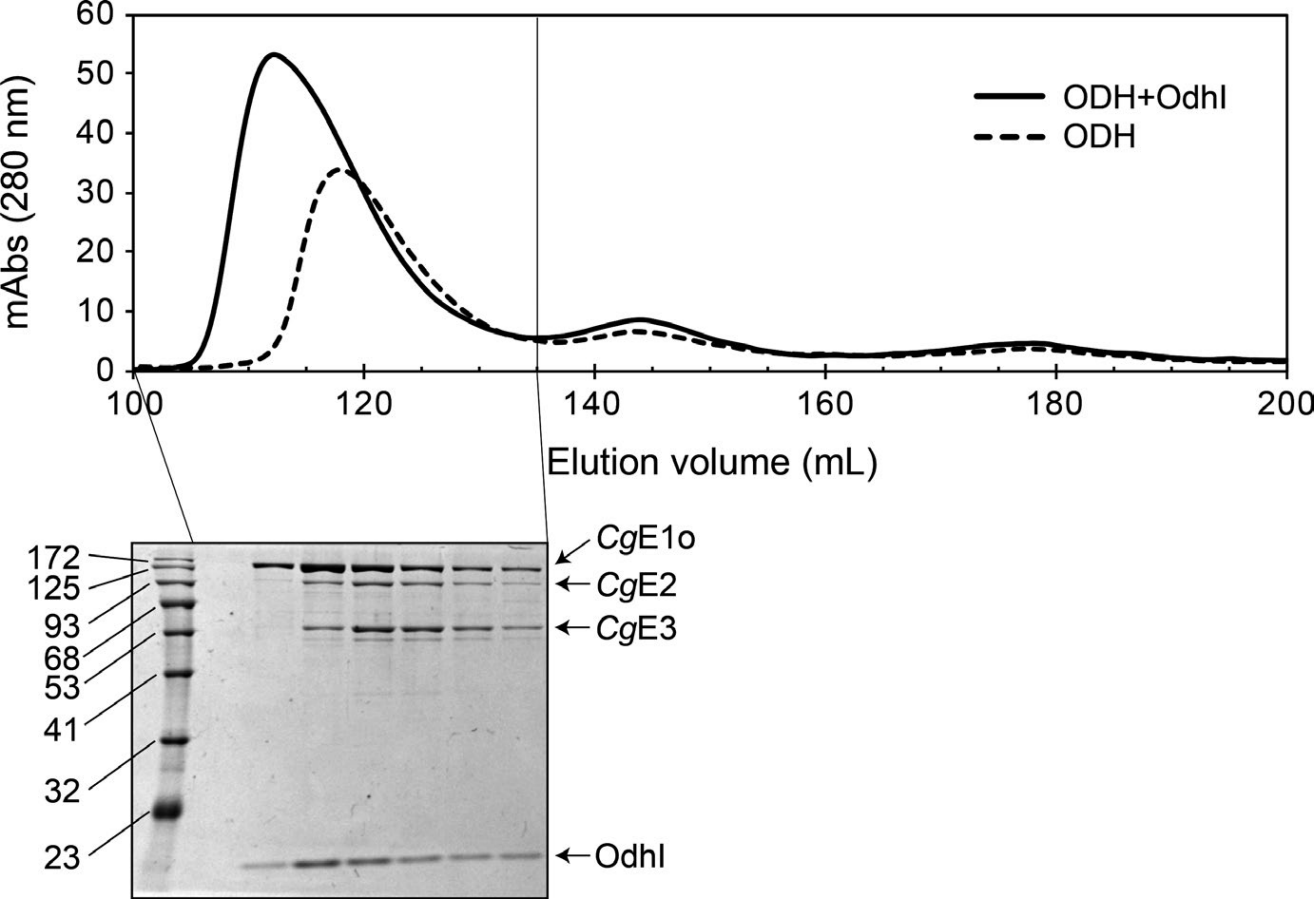
Effect of OdhI on the ODH complex. ODH complex was reconstituted by incubating *Cg*E1o (4.0 mg) with *Cg*E2‐E3 subcomplex (3.1 mg) on ice for 30 min. OdhI protein (4.6 mg) was added and further incubated for 30 min. The mixture was analyzed using gel filtration chromatography with a Superdex 200 column (upper). Aliquots (5 μl) of fractions (5 ml each) indicated were analyzed using 12% SDS‐PAGE, and the CBB staining gel image is also shown (lower)

OdhI inhibits ODH by binding to *Cg*E1o (Krawczyk et al., 2010; Niebisch et al., 2006). However, it is unclear whether OdhI just associates with *Cg*E1o in the ODH complex or elicits the dissociation of *Cg*E1o from the full ODH complex to reduce the ODH activity. We examined the effect of OdhI on *in vitro* reconstituted ODH complex using gel filtration chromatography. It was observed that OdhI induced a peak shift in the gel filtration chromatogram and a substantial portion of OdhI coeluted with *Cg*E1o as well as *Cg*E2‐E3 (Figure 6). This result suggested that OdhI did not dissociate *Cg*E1o from the ODH complex.

## 4 DISCUSSION

In the present study, we performed a detailed molecular characterization of PDH and ODH from *C*. *glutamicum in vivo* and *in vitro*. Using ultracentrifuge analysis, we detected the PDH and ODH of *C*. *glutamicum* in the fractions much smaller than those of *E*.* coli* (Figure 1). It was shown that the catalytic domains of *Azotobacter vinelandii* and *E*. *coli* E2p, as well as *E*. *coli* E2o, are assembled in 24‐mer structures using gel filtration (Knapp et al., 1998; Schulze et al., 1991). On the other hand, *Cg*E2 likely existed in a hexamer in the *Cg*E2‐E3 subcomplex, which is one fourth of the octahedral PDH and ODH complexes, according to our gel filtration analysis (Figure 3). The *in vivo* evidence and *in vitro* evidence indicate that PDH and ODH of *C*.* glutamicum* are small and compact compared with those of other species.

**FIGURE 7:**
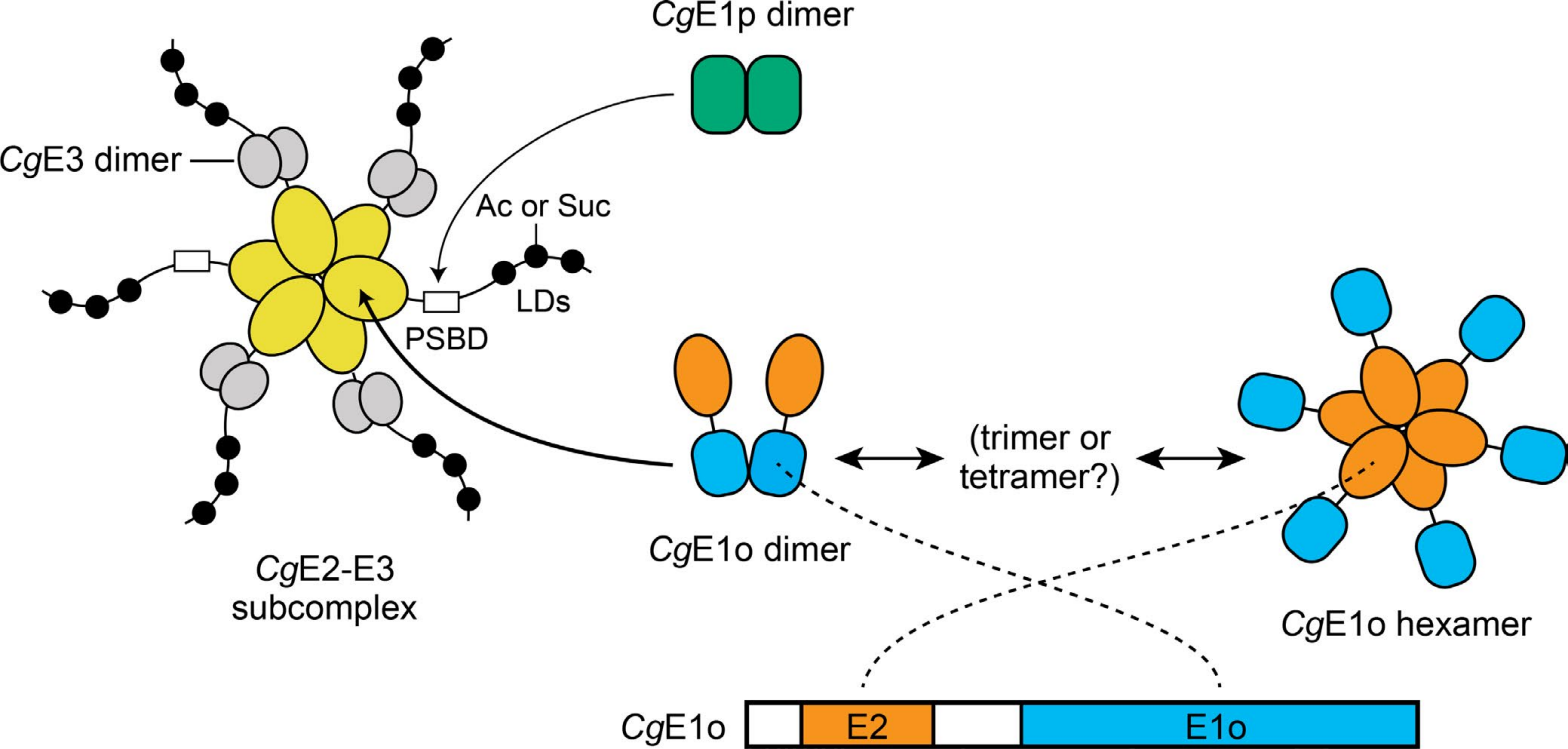
A current schematic model of the PDH‐ODH hybrid complex of *Corynebacterium glutamicum*. The core *Cg*E2‐E3 subcomplex is hypothetically composed of two *Cg*E2 (yellow) trimers and four *Cg*E3 (gray) dimers. *Cg*E1p (green) weakly associates with the PSBD of *Cg*E2 to form the PDH complex. *Cg*E1o stably exists in a hexamer and associates with the *Cg*E2 core potentially as the dimer to form the ODH complex. The hexamer and dimer of *Cg*E1o are putatively assembled via the E2 (orange) and E1o (blue) domains, respectively. *Cg*E1p and *Cg*E1o utilize lipoyl domains (LDs) on *Cg*E2 for tethering the acetyl group (Ac) derived from pyruvate and the succinyl group (Suc) derived from 2‐oxoglutarate, respectively, which are then transferred to CoA

A current schematic model of the *Cg*PDH‐ODH hybrid complex is shown in Figure 7. The core‐forming *Cg*E2‐E3 subcomplex (the predicted mass of 831 kDa) is hypothetically composed of two *Cg*E2 trimers and four *Cg*E3 dimers, in which six PSBD sites are available for E1 and E3 subunit binding. Four PSBD sites of *Cg*E2 are occupied by the *Cg*E3 dimer, and the remaining two sites are available for the *Cg*E1 binding. Though we were not successful in detecting a PDH complex using gel filtration analysis, it seems reasonable that PDH activity was saturated with 150 pmol of *Cg*E1p and 18 pmole of *Cg*E2‐E3 (Figure 5a). Considering that 18 pmol of *Cg*E2‐E3 contains 36 pmol of vacant PSBD sites, to which 72 pmol of *Cg*E1p monomer (36 pmol of the dimer) can bind, our experimental data support our hypothesis on *Cg*E2‐E3 stoichiometry. On the other hand, we successfully reconstituted the active ODH complex (Figure 2). Unlike *Ec*E1p, *Ec*E1o directly interacts with the E2 core, not the PSBD, via its N‐terminal extended region (Frank et al. 2007; Packman & Perham, 1986). The N‐terminal region of *Cg*E1o has a structural similarity to the N‐terminal region of *Ec*E1o, suggesting its key function in the association of *Cg*E1o with the hybrid complex (Hoffelder et al., 2010; Niebisch et al., 2006). From the estimated molecular mass (940 kDa) of the ODH complex, it is likely speculated that one or two molecules of *Cg*E1o (theoretical molecular mass of 135 kDa) associate with a single *Cg*E2‐E3 subcomplex at most. *Cg*E1o alone was present mainly in a hexamer in solution and equilibrium between multimer forms (Figure 3b). *Mycobacterium smegmatis* SucA (KGD), which has a high similarity to *Cg*E1o, is reported to be a dimer in the crystal structure (Wagner et al., 2011, 2014). Taking the above observations into consideration, it can be speculated that *Cg*E1o associates with the *Cg*E2 core as a dimer to form the active ODH complex. The ODH activity was not saturated even when 600 pmol of *Cg*E1o (300 pmol of *Cg*E1o dimer) was added to 18 pmol of *Cg*E2‐E3 (Figure 5a); an excess amount of *Cg*E1o dimer were bound to the binding sites on the *Cg*E2 core. We currently do not know the reason for the unsaturated ODH activity with the amount of *Cg*E1o and need to study this further to understand the relationship between the molecular assembly and activity of *Cg*ODH.

We observed *Cg*E1o‐ and *Cg*E1p‐dependent decrease in PDH and ODH activities, respectively (Figure 5b,c). This evidence is consistent with the formation of the PDH‐ODH hybrid complex, as suggested previously (Hoffelder et al., 2010; Niebisch et al., 2006), in which both *Cg*E1p and *Cg*E1o subunits associate with the single *Cg*E2‐E3 subcomplex. As mentioned above, in *E*.* coli*, E1p and E1o utilize different sites (PSBD and the core, respectively) to bind to the cognate E2 (Packman & Perham, 1986; Frank et al., 2007; Arjunan et al., 2014), and *Cg*E1p and *Cg*E1o would not compete for binding to *Cg*E2. Both *Cg*E1p and *Cg*E1o utilize lipoyl residues on *Cg*E2 for tethering the acetyl and succinyl group derived from pyruvate and 2‐oxoglutarate, respectively. We thus speculated that *Cg*E1p and *Cg*E1o might compete for the availability of lipoyl residues on *Cg*E2.

Since the active ODH complex was retained during gel filtration, but PDH was not (Figure 2, Figure A2), we assumed that the association of *Cg*E1o with *Cg*E2‐E3 was more stable than that of *Cg*E1p with *Cg*E2‐E3. Since the association of E1p with E2 is retained in gel filtration in other bacteria (Lessard et al., 1998; Schulze et al., 1993), *Cg*PDH is likely more fragile compared to other PDHs. *C*. *glutamicum* PDH showed a *K*_m_ value of 3.5 mM for pyruvate (Figure 4), which is comparable to the intracellular concentration (7.5 mM) of pyruvate reported in *E*. *coli* (Yang et al., 2001). Since the *Cg*PDH complex seemed more fragile compared to *Cg*ODH, and PDH activity was more strongly affected by *Cg*E1o than ODH activity was by *Cg*E1p, the PDH activity might be altered in response to the pyruvate levels and the expression levels of *Cg*E1o. Conversely, the *K*_m_ value of *Cg*ODH for 2‐oxoglutarate (0.01 mM) was much lower than the intracellular concentration of 2‐oxoglutarate (0.44 mM) (Bennett et al., 2009) and the *Cg*ODH complex was stable. The ODH activity may be unperturbed by the 2‐oxoglutarate levels, and the availability of *Cg*E1o and OdhI was perhaps critical to control the activity. The *V*_max_ values of PDH and ODH were much lower than those previously reported using *C*.* glutamicum* lysates (148 and 134 nmol min^−1 ^mg^−1^ for PDH and ODH, respectively) (Hoffelder et al., 2010). We assumed that it is because we used the NAD^+^ analog (APAD^+^) in enzyme assays, which, in some cases, give different specific activities from those with NAD^+^ (Kavanagh et al., 2007).

The regulation of PDH and ODH activities is a key event in glutamate overproduction in *C*. *glutamicum*. The OdhI‐dependent inhibition of ODH is well characterized as a critical determinant for glutamate production (Kim et al., 2010; Schultz et al., 2007), and the decrease in the PDH activity is observed in glutamate‐producing conditions (Hasegawa et al., 2008). Considering two enzyme activities on a single complex, the change in the subunit ratio of *Cg*E1p and *Cg*E1o would affect the balance of PDH and ODH activities. In this regard, it was intriguing that the shift of *Cg*E2 toward higher molecular mass fractions was observed in glutamate‐producing conditions (Figure 1b). This may reflect the association of OdhI with the ODH (or hybrid) complex (Figure 6). Alternatively, the change in the molecular assembly and/or stoichiometry of the hybrid complex could occur, which would contribute to the control of PDH and ODH activities during glutamate overproduction.

We found that *Cg*E1o alone formed multimers, mainly a hexamer, which were ready to form an active ODH complex (Figure 3). The *in vitro* multimerization of *Cg*E1o agreed with the *Cg*E1o detected in higher molecular mass fractions, independent of *Cg*E2, *in vivo* (Figure 1b). The E1o component is usually a dimer (Frank et al., 2007; Wagner et al., 2011). However, considering that *Cg*E1o has an extra E2‐catalytic domain capable of forming a trimer (Izard et al., 1999; Knapp et al., 1998; Mattevi et al., 1992, 1993; Wang et al., 2014), following possibilities for the detected hexamer were considered: three *Cg*E1o dimers and two *Cg*E1o trimers, and we prefer to consider the possibility of two *Cg*E1o trimers assembled via the extra E2 domain (Figure 7). A structural study needs to be conducted to reveal the subunit assembly of the *Cg*E1o hexamer. Meanwhile, *Cg*E1p existed majorly as a dimer *in vitro* (Figure A2), and it also migrated to higher molecular mass fractions independent of *Cg*E2 *in vivo* (Figure 1b). It might be speculated that *Cg*E1p interacted with unknown proteins *in vivo*.

## 5 CONCLUSION

This study revealed the unique molecular architecture of *C*.* glutamicum* PDH and ODH. They can form compact‐sized PDH‐ODH hybrid complexes. At present, we are not sure whether all complexes were a hybrid form of PDH and ODH *in vivo*. Thus, we speculated that some might exist as independent PDH or ODH complexes. Our present study also revealed the fragility of the *Cg*PDH complex and the unusual multimerization of *Cg*E1o, which has not been reported. The compact size of the hybrid complex would provide an advantage to determine its whole structure and understand the molecular mechanism to balance the PDH and ODH activities, which should be critical to control the metabolic flux in *C*.* glutamicum*.

## CONFLICT OF INTEREST

None declared.

## AUTHOR CONTRIBUTIONS

**Hirokazu Kinugawa:** Formal analysis (equal); investigation (equal). **Naoko Kondo:** Formal analysis (equal); investigation (equal). **Ayano Komine‐Abe:** Formal analysis (supporting); investigation (supporting). **Takeo Tomita:** Methodology (equal). **Makoto Nishiyama:** Supervision (lead); writing – review & editing (equal). **Saori Kosono:** Conceptualization (lead); funding acquisition (lead); methodology (equal); project administration (lead); validation (lead); writing – original draft (lead); writing – review & editing (equal).

## ETHICS STATEMENT

None required.

## Data Availability

All data generated or analyzed during this study are included in this article.
